# 9. The Skip Phenomenon in *Staphylococcus aureus* Bacteremia: Clinical Associations

**DOI:** 10.1093/ofid/ofab466.009

**Published:** 2021-12-04

**Authors:** John Raymond U Go, Larry M Baddour, Brian Lahr, Muhammad R Sohail, Raj Palraj

**Affiliations:** 1 Mayo Clinic Rochester, Rochester, MN; 2 Mayo Clinic College of Medicine, Rochester, MN; 3 Mayo Clinic, Rochester, Minnesota; 4 Baylor College of Medicine, Houston, Texas

## Abstract

**Background:**

Serial blood cultures are integral in managing *Staphylococcus aureus* bacteremia (SAB) as clinicians rely on the results to determine infectious complication risks and antibiotic duration. Current IDSA guidelines suggest a single set of negative blood cultures is adequate evidence of SAB clearance. Several studies, however, have identified the skip phenomenon (SP), which is the occurrence of intermittent negative blood cultures, and have recommended obtaining additional blood cultures to document bacterial clearance (Table 1). We therefore examined patients who manifested the SP to determine its clinical significance and to study this, associations were tested for SP in relation to various baseline factors as well as clinical outcomes.

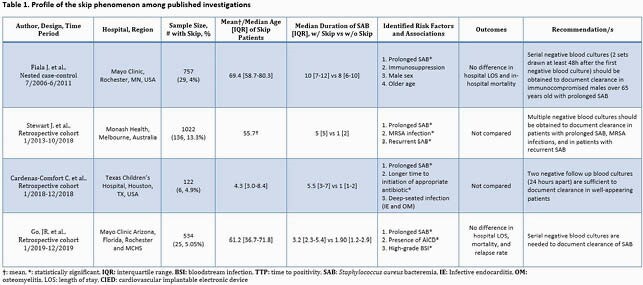

**Methods:**

We performed a retrospective, multicenter study of all patients with a positive blood culture for *S. aureus* from January 2019 to December 2019 using data collected from electronic health records and the clinical microbiology laboratory.

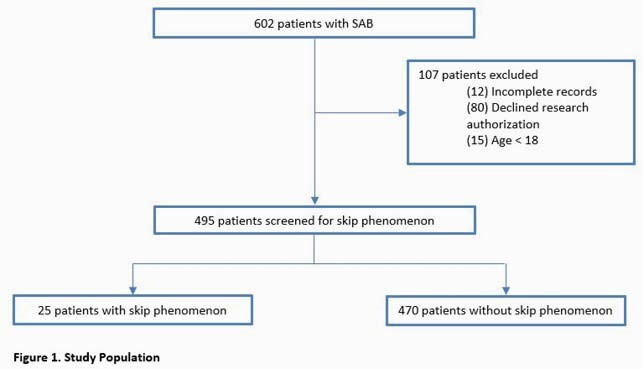

**Results:**

A total of 602 patients with SAB were identified and 495 patients were included in the investigation (Figure 1). Overall, 25 (5.1%) patients had the SP. Significant differences between those who did and did not manifest the SP included higher rates of injection drug use, automatic implantable cardioverter defibrillator, and community onset of infection in the SP cohort (Table 2). Moreover, the median duration of SAB was longer (3.2 [2.3-5.4] vs 1.90 [1.2-2.9] days, p=0.002), and high-grade SAB, (88.0% vs 58.7%, p=0.004), complicated bacteremia (92.0% vs 67.9%, p=0.011) and IE diagnosis (28.0% vs 11.3%, p=0.013) were all more common in the SP group. In unadjusted outcome analyses, association of SP with hospital length of stay was not significant, although a higher risk of in-hospital mortality among SP patients approached statistical significance (p=0.055). Analysis of 435 hospital survivors revealed no significant differences in rates of 1-year mortality or 90-day relapse between the two groups (Table 3).

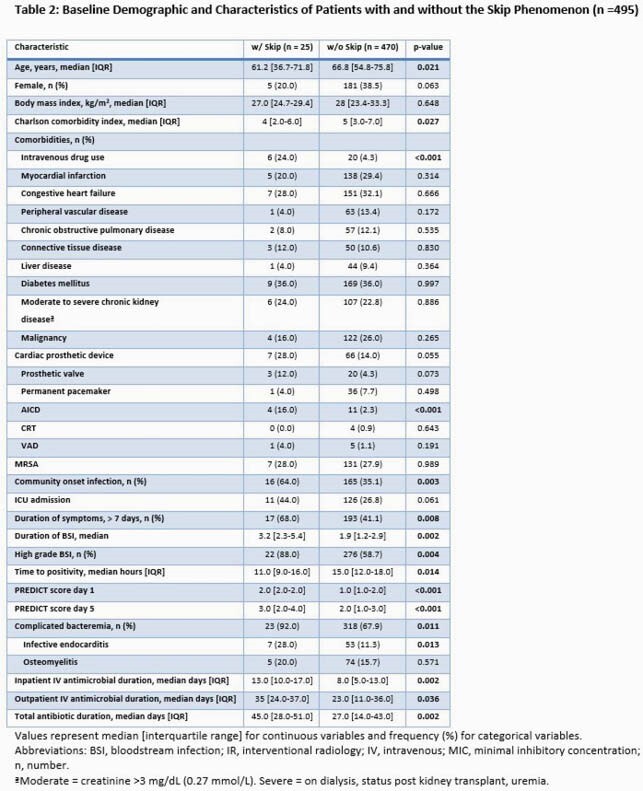

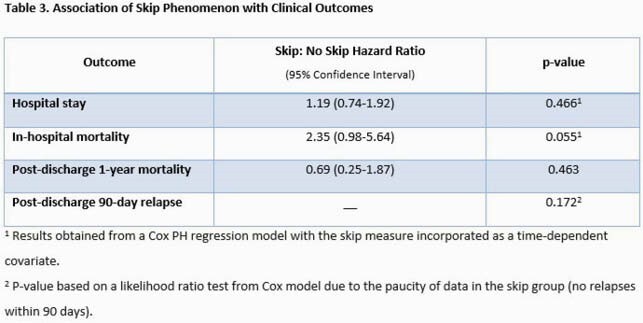

**Conclusion:**

Findings of the current investigation demonstrates an increased risk of SAB complications in patients with the SP and support the notion that serial negative blood cultures are needed to document clearance of SAB.

**Disclosures:**

**Larry M. Baddour, MD**, Boston Scientific (Individual(s) Involved: Self): Consultant; Botanix Pharmaceuticals (Individual(s) Involved: Self): Consultant; Roivant Sciences (Individual(s) Involved: Self): Consultant **Muhammad R. Sohail, MD**, **Medtronic** (Consultant)**Philips** (Consultant)

